# The Gallivanters’ Folly

**Published:** 1885-07

**Authors:** Ben. H. Pratt


### THE BISTOURY.

Vol. XXI. ELMIRA, N. Y., JULY, 1885. No. 2.

### Contributions.

### THE GALLIVANTERS' FOLLY.

BEN. H. PRATT.

If one were asked for an honest, square-toed reason—or rather, if one set out with a determination to give his or her honest reason—for taking a summer jaunt to, or making a summer sojourn at, the springs, the sea shore, the mountain or elsewhere, what queer causes some of us would make declaration of!

Modern notions have changed very much in the matter of recreation, or rest, or pastime, or recuperation. It is a comparatively late thing for people to trundle off somewhere every summer. It is a craze, but one that is destined to become chronic, because anything that necessitates the spending of time and the spending of money becomes the standard of comparative financial eminence which is the present respectometer of society.

Setting aside the grand, general reason for going off somewhere, to wit: the demonstration to all the world—our little world of society—that we have the wherewithal to enable us to leave our accustomed avocations and idle away a month, two months, three months or more—aside from this, which all must grant—what is the reason which we each give ourselves, in the satisfying of our own consciences, for the going, the gallivanting, the idling? Of course no one is foolish enough to admit—to himself or herself—that the above reason is *the* reason in our case. Then let's see what these reasons are.

For our own health. That's the first one that comes into our minds. We all have something the matter with us. Almost any one can trump up something in the way of a malady. We all have a physical weakness somewhere about us, or can readily persuade ourselves that we have. It is nobody's business but our own, of course, and it isn't necessary to proclaim it. No one ever disputes a man or woman who declares that they have a misery. It isn't polite, and then, we may want people to believe, some time or other, that we have a physical difficulty of some sort, that needs going away for. This is a reason that any one can give, and believe he is honest in giving it, for making his little summer go a perfectly legitimate thing to be undertaken. We have our go because we are somewhat poorly.

For a good rest. There are some who have not yet summoned up the courage to admit that they are sick, or even ailing. They don't show it in their faces nor in their movement, and haven't the gall to claim it. Such, however, can easily persuade themselves that they are tired out, run down, have too much of a strain upon them and wouldn't last through another year without a season of rest. They work up their own imaginations to the desired point, and off they go. Rest is a good thing, if one rests judiciously, and everyone is supposed to be the best judge of his own mode or method of resting. Nobody has any right to dictate to another how he or she shall rest, except, of course, that nobody can possibly rest at home. Who ever thought of taking a summer's rest at home! That would be preposterous. So we go away to rest. Anywhere is a resting place—away from home—but generally the farther away from home the more restful is the restfulness.

But the most of us go to recuperate and recreate. There is a biologic mystery extant which must ever remain such. Science informs us that certain intensified muscular or nervous activity consumes certain elements of vitality, and that these elements can never be restored to us again except by a complete abandonment of our mental or physical energies and activities. We must separate ourselves wholly from all customary occupations, cease to think of them, and allow nature to remake what we have been using up. To give one's self over, then, to absolute inanition, make no effort whatever, only do and be that which becomes done of the mind's or the body's own involuntariness—that is the conventional rest of the period. One can't so rest at home. Certainly not. One must go away and disassociate himself not only from every care and responsibility, but from all the persons and things that even for a moment can remind him that he has ever had or ever will have anything to do or to think about. This is recreation theorized down to scientifico-medico-hygienic dots, according to the latest pronunciamentos of the most astute hygienists of uppertendom.

Of course there are people who do, really and truly, go away for their health. There are, probably, certain forms of disease that may possibly be treated with more success in one climate than in another. And there are certain other forms of disease which might possibly be alleviated by a change of scene, such as maladies of the mental or nervous organism. It is doubtless true that it is often easier to find absolute quiet and rest away from home, amid the seclusion of the country, for instance, or upon the unfrequented mountain heights. Then there are doubtless people who are really overworked and need a complete rest, and feel that the temptation and the incentive to work, while at their homes, would be such as could not be resisted, and for such, departure is essential. It cannot be denied, either, that a change of habitation will often restore the really jaded energies, and actually aid the recreation, the making over again which nature does certainly effect under certain circumstances. There always have been some people who beyond question have been and are being helped by a longer or shorter sojourn from home amid just the proper environment. These do not need to ask themselves why they go away, and the wisdom of their going has been repeatedly demonstrated. But they are not one per cent. of the summer's gallivanting multitudes.

But of the multitude that flits away to the summer resorts about this time of the year, how many really have any reason for going save that they are tired of being at home, or go because it is the proper thing to do in order to maintain a correct standing in society, or to have a good time with somebody they may meet, or to get way from the surveillance of those who know them far too well to allow any indiscretion to remain unheralded to the world, or because they have nothing else to do, or to display a grand array of toilets, or for flirtation's sake, or for the scores of reasons which the reader can mentally supply without assistance from the writer? The number who thus go and dawdle the summer away, and have no good reason for going, is legion. Some may convince themselves that they have a reason for wasting a fourth or a third of every year of their lives in this way, but they do not and can not deceive others. The periodical returns of poor health and physical exhaustion are too regular to be natural, and people would much more favorably impress those who know them if they would confess that their going is honestly for fun, or in compliance with a custom of bon-ton society. This trumping up of sham excuses for going to summer resorts is one of the worst features of the craze, and ought to be frowned down instead of upheld by people of reputed sense. The resort to a deception to cover up a folly only makes the folly the more glaring. Besides, the custom is going to work harm some of these days.

The fact that nearly everybody of any pretension whatever to social position does go to summer resorts during the season, is one to be looked upon with considerable anxiety. Many go who ought not to go; who cannot afford it; who do so at the expense of their creditors, or at the sacrifice of actual home comforts; who are driven to the most desperate straits to achieve the fashionable boon; who would deny themselves almost anything, and submit to almost anything, to keep their places in the race for social position. It keeps many poor who might be comfortably off at home, if not reasonably affluent. Many, with sufficient means to go and not cheat anybody by their going, nevertheless often sacrifice business and neglect grave duties to keep pace with the skurrying crowd of summer saunterers. Domestic affairs that absolutely demand attention are deserted in obedience to Mrs. Grundy's behests. Children and homes are often neglected, and families cruelly separated by the silly compliance with the social requirement. The physicians will bear me out in the assertion that more people are physically injured than helped by the alleged seeking after health upon these summer jaunts and sojournings. Inconveniences are submitted to without any complaining whatever, that would not be tolerated for a day at home. Many who live not only comfortably but luxuriously at home, are often compelled to endure sore impositions if not downright misery at these crowded places of resort to which the fashionable feel that they must rush, and in which they must abide, be the circumstances as distressing as they may. Those who go to these places know precisely how true these things are and if honest they will admit their truth.

This summer flitting is a demoralizing process. It affects the health and spoils the disposition. A sacrifice of bodily comforts, the yielding up of accustomed home conveniences for the wretched accommodations of the best of the crowded summer resorts, beget a state of mind that cannot be anything but prejudicial. One frets and fumes over his little, cramped up, overheated quarters at a crowded hotel, and contrasts his roomy home therewith, to the detriment of the former. He longs to return long before fashion says he may, but the edicts of society are imperative and he must remain and endure. Then come the rounds upon rounds of dissipation to which one is tempted to yield. These exercise his physical powers beyond the limits of healthful indulgence, and strain his mental capacity in a like ratio. There is nothing that will kill so quickly and so unconsciously the unhappy victim as will dissipation. People return from their summer sprees with constitutions broken down, with habits of business and domestic routine all demoralized, and not unfrequently with their moral sense forever blunted. This summer dissipating impoverishes the pocket, the mind, the health, the disposition, the heart. It is one of the worst habits of the American people. And it is a growing one. The number of those who live upon this habit is said to constitute twelve and a half per cent. of the population of the state of New York. Perhaps the ratio in Pennsylvania is less, and I sincerely hope it is. But the craze is becoming as furious here as anywhere, and is limited only by the sturdy element that fortunately stands as the basis of our social economy. One would suppose that a custom so obviously demoralizing, and one that is so plainly enervating, would soon be abandoned to the weak-minded and physically impotent. Perhaps we shall have to await a great social crisis before we realize the issue to which all this is leading. There surely must come such a crisis if the rage for this inane custom continues much longer. It is not necessary to convince people that they are happier and better in every way at home than abroad during the dog-days, for they already admit this when they give their honest opinion, but the aim should be to impress people with the loftiness of preserving their self-respect by letting the fools do the summer gallivanting.

